# A Comparison of Magnetic Resonance Imaging Assessment and Biopsy Outcomes with and Without Central Review in Two Swedish Regional Organized Prostate Cancer Testing Programs

**DOI:** 10.1016/j.euros.2025.05.008

**Published:** 2025-06-05

**Authors:** Jonas Wallström, Max Alterbeck, Rebecka Arnsrud Godtman, Ola Bratt, Thomas Jiborn, Erik Thimansson

**Affiliations:** a Department of Radiology, Institute of Clinical Sciences, Sahlgrenska Academy, University of Gothenburg, Gothenburg, Sweden; b Department of Radiology, Sahlgrenska University Hospital, Gothenburg, Sweden; c Department of Translational Medicine, Urological Cancers, Lund University, Malmö, Sweden; d Department of Urology, Skåne University Hospital, Malmö, Sweden; e Department of Urology, Institute of Clinical Sciences, Sahlgrenska Academy, University of Gothenburg, Gothenburg, Sweden; f Department of Urology, Sahlgrenska University Hospital, Gothenburg, Sweden; g Department of Urology, Helsingborg Hospital, Helsingborg, Sweden; h Department of Translational Medicine, Diagnostic Radiology, Lund University, Malmö, Sweden; i Department of Radiology, Helsingborg Hospital, Helsingborg, Sweden

**Keywords:** Prostatic neoplasms, Early detection of cancer, Magnetic resonance imaging, Observer variation

## Abstract

**Background and objective:**

The European Council advises evaluating the feasibility of organized prostate cancer testing (OPT) programs, but it is unclear whether results from screening trials can be replicated in population-based testing. The aim of this study is to compare magnetic resonance imaging (MRI) assessments and biopsy outcomes with and without a central review in two Swedish OPT programs.

**Methods:**

Two regional population-based OPT programs invited 65 000 men (2020–2022). MRI scans were read locally, and biopsies followed a strict MRI-based and prostate-specific antigen (PSA) density–based protocol. A blinded central review was done by two radiologists with 8 and 9 yr of experience. Reader agreement was assessed with percentages and kappa scores. Positive predictive values (PPVs) for detecting grade group (GG) 2–5 prostate cancer were calculated with 95% confidence intervals (CIs).

**Key findings and limitations:**

MRI scans for 416 men (median age 52 yr) with PSA ≥3 ng/ml were evaluated. In Skåne, 27% of scans were primarily assigned Prostate Imaging Reporting and Data System (PI-RADS) scores ≥4, compared with 10% in Västra Götaland. At the primary reading, 76 men had PI-RADS ≥4, yielding 43 GG 2–5 prostate cancer cases: PPV 0.57 (95% CI 0.45–0.67). At the central review, 65 men had PI-RADS ≥4. Out of 61 men biopsied, 50 had GG 2–5 prostate cancer: PPV 0.82 (95% CI 0.71–0.90, *p* < 0.001 for PPV difference). The central review radiologists’ kappa score was 0.83. No additional biopsies were taken based on the central review findings.

**Conclusions and clinical implications:**

In population-based screening with local MRI reading, MRI assignment may vary substantially. Centralized reading could reduce these differences and increase the biopsy PPV for GG ≥2 cancer.

**Patient summary:**

In this report, we reviewed local magnetic resonance imaging (MRI) reading in population-based screening. We found that MRI assignment varied between centers. We conclude that centralized reading could reduce differences and improve biopsy outcomes.

## Take Home Message

In this report, we reviewed local magnetic resonance imaging (MRI) reading in population-based screening. We found that MRI assignment varied between centers. We conclude that centralized reading could reduce these differences and improve biopsy outcomes.

## 1 Introduction

Since December 2022, the European Council recommends its member states to evaluate the feasibility and effectiveness of organized prostate cancer (PC) testing (OPT) programs [1]. The European PRAISE-U consortium aims to reduce morbidity and mortality of PC through risk-based screening programs [2]. The first two Swedish population-based, regional OPT programs started in 2020 [1,3]. Since November 2024, OPT programs are ongoing in 16 regions and 208 794 men had been invited. In these programs, testing is based on prostate-specific antigen (PSA) and magnetic resonance imaging (MRI). Results from the first invitation of 68 000 50-yr-old men showed substantial intraregional variations in Prostate Imaging Reporting and Data System (PI-RADS) distributions and the proportion of diagnosed low-grade cancer cases [4].

It is fundamental to ensure high-quality diagnostics for population-based screening to be effective and equal. Unlike several major screening trials where radiology and pathology have been concentrated in high-volume centers, Swedish OPT is carried out in decentralized standard care. Given the known interobserver variability in PI-RADS and PC grading [[5], [6], [7]], it is crucial to examine the diagnostic outcomes in such a decentralized diagnostic setting. In particular, the assessment of MRI in screening presents specific challenges due to the inclusion of relatively young men with a lower probability of cancer. Many younger men have smaller, denser prostates and inflammatory signal changes [8], which may lead to diagnostic errors—both missing cancer cases and overcalling benign lesions.

The main objectives of this study were to expose any relevant differences in PI-RADS assessments and biopsy outcomes between two large regional population-based OPT programs and to evaluate whether a central review of MRI scans can reduce inter-regional differences and improve the positive predictive value (PPV) of prostate biopsy to detect clinically significant PC.

## 2 Patients and methods

### 2.1 Study population and diagnostic pathway

The overreaching structure and details of the OPT programs in Sweden have been described elsewhere [1,4].

During the years 2020–2022, men born in 1958, 1964, 1966, 1970, 1971, and 1972 were invited in Region Skåne and men born in 1970, 1971, and 1972 were invited in Region Västra Götaland.

The diagnostic pathway included MRI for all men with a PSA value above 3 ng/ml.

MRI was performed at eight public hospitals in Skåne and at six public hospitals in Västra Götaland. In Skåne, radiologists engaged in OPT were organized into three centers, each responsible for several MRI departments. The radiologists who performed the central review were primary readers in a subset of MRI scans performed at two centers.

In both regions, 3 or 1.5 T MRI was performed without contrast enhancement and assessed in accordance with the PI-RADS v2.1 document. Protocol and scanner details have been described previously [1]. No formal requirements or certifications were used for selection of radiologists; participating radiologists were general radiologists with an average of 5 yr (range 2–8 yr) of experience of prostate MRI reading and most had completed national and international training in the field.

It was mandatory for the radiologists to register and report using a national electronic template. The template provides a structured report and facilitates automated histological feedback from biopsy results with a correlation between localization of MRI lesions, cores, and histology [9].

**Fig. 1:**
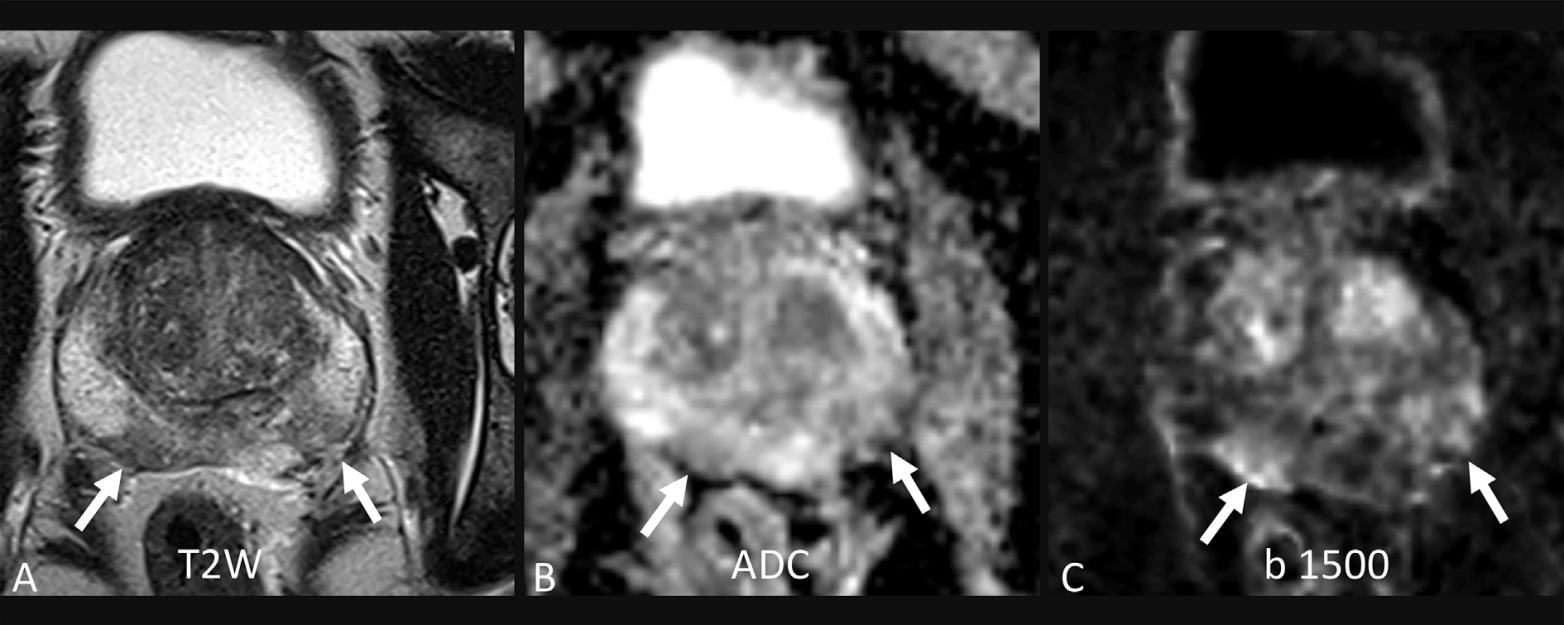
Siemens Healthcare AG (Erlangen, Baviaria, Germany) VIDA OPT prostate MRI (3 T) with (A) bilateral T2W hypointensities and (B and C) mildly restricted diffusion with ADC values >1200 × 10^–6^ mm^2^/s. The primary reader assigned bilateral PI-RADS 4 lesions; both central review readers assigned PI-RADS 2. Two rounds of bilateral targeted biopsies (six cognitive biopsies in the first round and eight TRUS/MRI fusion biopsies in the second round) showed benign prostate tissue only. A repeat MRI scan 2 yr later in the OPT program was assessed to be PI-RADS 2. ADC = apparent diffusion coefficient; MRI = magnetic resonance imaging; OPT = prostate cancer testing; PI-RADS = Prostate Imaging Reporting and Data System; TRUS = transrectal ultrasound; T2W = T2 weighted.

Predefined MRI quality indicators in the two regional OPT programs included proportions of positive (PI-RADS 4–5) and negative (PI-RADS 1–3) MRI. PI-RADS scores were further correlated with the diagnosis of PC on subsequent biopsy (grade group [GG] 1 vs GG 2–5). An example of image quality is provided in Fig. 1.

### 2.2 Biopsy indications, biopsy technique, and pathohistological assessment

Biopsy indications were a PI-RADS ≥4 lesion or PI-RADS 1–3 lesions combined with PSA density ≥0.15 ng/ml^2^. Biopsies were taken at seven public hospitals in Skåne and in six in Västra Götaland. In Västra Götaland, only targeted biopsy (with cognitive fusion) was performed for PI-RADS 4–5 lesions and PSA density <0.15 ng/ml^2^, and combined targeted and systematic biopsies were performed in men with PI-RADS 4–5 and PSA density ≥0.15 ng/ml^2^. In Skåne, combined targeted and systematic biopsies were performed to varying degrees for PI-RADS 4–5 lesions, initially depending on PSA density and lesion size, but ultimately for all PI-RADS 4–5 lesions. There was also a higher utilization of image fusion software (transrectal ultrasound/MRI) in Skåne, as the largest center used this technique routinely. The biopsy specimens were assessed by three pathology departments in Skåne and four in Västra Götaland.

### 2.3 Central review of MRI scans

No additional biopsies were taken based on the central review findings.

Two radiologists subspecialized in prostate MRI, with 8 and 9 yr of experience, reviewed all scans independently. They were blinded to PSA levels, original referrals/reports, and biopsy outcomes. Scan-assigned discordant PI-RADS scores between the two central reviewers were discussed and resolved by a consensus assignment by the same two readers.

### 2.4 Data retrieval and ethical approval

All men were informed about the study by letter and given the option to not participate (opt out) according to the approval by the Swedish Ethical Review Authority (registry number 2022-02512-01). Data were retrieved from the regional OPT registries and medical records.

### 2.5 Statistical analysis

We studied MRI assessment and detection of GG 2–5 PC before and after the central review. The *p* values for the comparison of PPVs were based on 10 000 bootstrap samples, considering that the dependence between PPV estimates due to both the primary and the central review was performed for the same men. In each bootstrap sample, the rows where either the primary or the central reading (not both) was MRI positive (PI-RADS 4–5), the roles of the primary and central reading were swapped with a probability of 0.5, and then the difference in PPVs was calculated. By this, a null distribution for the difference in PPVs was achieved, and the observed difference was compared with this distribution to get a *p* value. A *p* value of <0.05 was considered statistically significant. Cohen’s kappa score with linear weights and 95% confidence intervals (CIs) were used to assess the agreement between expert readers. All analyses were performed with SPSS version 30 (SPSS Inc., Chicago, IL, USA) and R statistical software, version 4.2.3 (R Foundation for Statistical Computing, Vienna, Austria).

## 3 Results

The population characteristics, distributions of PI-RADS scores, and biopsy outcomes in the two regions are summarized in Table 1.

**Table 1:** Age of the studied population, and MRI and biopsy characteristics a

	Skåne (*N* = 210)	Skåne 50-yr old (*N* = 105)	Västra Götaland (*N* = 206)
Age (yr)	53.7 (50–56)	50 (50–50)	50 (50–50)
PSA (ng/ml)	3.85 (3.29–5.0)	3.74 (3.24–4.78)	3.90 (3.33–4.95)
Prostate volume (ml)	35 (29–44)	35 (27–40)	38 (28–47)
PSA density (ng/ml^2^)	0.11 (0.09–0.16)	0.12 (0.09–0.16)	0.08 (0.12–0.15)
MRI	210	105	206
PI-RADS 1–2	108 (51)	55 (52)	143 (69)
PI-RADS 3	46 (22)	25 (24)	43 (21)
PI-RADS 4	41 (20)	19 (18)	11 (5)
PI-RADS 5	15 (7)	6 (6)	9 (4)
Biopsy	89	44	57
Benign	40 (45)	28 (64)	16 (28)
GG 1 cancer	6 (7)	1 (2)	14 (25)
GG 2 cancer	31 (35)	11 (25)	12 (21)
GG 3 cancer	6 (7)	3 (7)	10 (18)
GG 4–5 cancer	6 (7)	1 (2)	5 (9)

GG = grade group; IQR = interquartile range; MRI = magnetic resonance imaging; PI-RADS = Prostate Imaging Reporting and Data System; PSA = prostate-specific antigen.

a Data are presented as *n*, *n* (%), or median (IQR).

The median age was 53.7 yr in Skåne and 50.0 yr in Västra Götaland, reflecting differences in the invited age groups. The PSA levels and prostate volumes in men in Skåne aged 50 yr were similar to those in men in Västra Götaland (Table 1).

### 3.1 Primary reading and biopsy results

Nine men opted out of the study, leaving 416 MRI scans for an analysis, 210 in Skåne and 206 in Västra Götaland. PI-RADS ≥4 was assigned to 56/210 scans (27%) in Skåne and to 20/206 scans (10%) in Västra Götaland.

Most PI-RADS 5 lesions yielded at least GG 2 cancer on biopsy (Table 2): 13/15 (87%) men in Skåne and seven of nine (78%) men in Västra Götaland. Cancer detection for PI-RADS 4 differed, with more benign biopsy results (49% vs 9%) and fewer GG 1 cancer cases (7% vs 45%) in Skåne than in Västra Götaland.

**Table 2:** Cross-tabulation of primary reader MRI scores and biopsy results in Skåne and Västra Götaland a

	Skåne				Västra Götaland			
	PI-RADS 2 (*n* = 228)	PI-RADS 3 (*n* = 46)	PI-RADS 4 (*n* = 41)	PI-RADS 5 (*n* = 15)	PI-RADS 2 (*n* = 143)	PI-RADS 3 (*n* = 43)	PI-RADS 4 (*n* = 11)	PI-RADS 5 (*n* = 9)
No biopsy	90 (83)	31 (67)	0	0	125 (88)	24 (56)	0	0
GG 1	2 (2)	1 (2)	3 (7)	0	6 (4)	2 (5)	5 (45)	1 (11)
GG 2	6 (6)	6 (13)	12 (29)	5 (33)	1 (1)	7 (16)	3 (27)	1 (11)
GG 3	0	0	3 (7)	3 (20)	2 (1)	2 (5)	2 (18)	4 (44)
Gleason GG 4–5	0	0	3 (7)	5 (33)	1 (1)	2 (5)	0	2 (22)
Benign	10 (9)	8 (17)	20 (49)	2 (13)	8 (6)	6 (14)	1 (9)	1 (11)

GG = grade group; MRI = magnetic resonance imaging; PI-RADS = Prostate Imaging Reporting and Data System.

a Data are presented as *n* (%).

In the group of men with PI-RADS 4–5 biopsy indication (Table 3), any PC was detected in 61% in Skåne and 90% in Västra Götaland. For GG 2–5 PC, the corresponding numbers were 55% and 60%. The numbers needed to biopsy (NNBs) to detect one case of GG 2–5 PC were 1.8 and 1.7, respectively.

**Table 3:** Number of cancer cases detected and positive predictive values by biopsy indication group in Skåne and Västra Götaland, based on PI-RADS scoring by primary readers

	Skåne			Västra Götaland		
Biopsy group	PI-RADS ≥4	PI-RADS 3 and PSAD ≥0.15	PI-RADS 1–2 and PSAD ≥0.15	PI-RADS ≥4	PI-RADS 3 and PSAD ≥0.15	PI-RADS 1–2 and PSAD ≥0.15
*n*	56	12	25	20	22	27
Biopsy, *n* (%)	56 (100)	11 (92)	18 (72)	20 (100)	19 (86)	18 (67)
Any PC, *n* (PPV %)	34 (61)	7 (64)	8 (44)	18 (90)	13 (68)	10 (56)
GG 2–5 PC, *n* (PPV %)	31 (55)	6 (55)	6 (33)	12 (60)	11 (58)	4 (22)
Number of men needed to biopsy to detect one GG 2–5 PC	1.8	1.8	3	1.7	1.7	4.5

GG = grade group; PC = prostate cancer; PI-RADS = Prostate Imaging Reporting and Data System; PPV = positive predictive value; PSAD = prostate-specific antigen density.

### 3.2 Central reading and biopsy result consequences

The agreement between the two expert readers performing the central review was very good, with a kappa score of 0.83 (95% CI 0.77–0.88). A comparison of the central review and the primary reports is shown in Table 4. Overall, the agreement between the central review and primary reports was 295/416 (71%). At the central review, the primary reading in Skåne was downgraded in 57 (27%) cases and upgraded in 16 (7.6%). In Västra Götaland, we found 26 (13%) cases with upgrading and 22 (11%) cases with downgrading.

**Table 4:** Cross-tabulation of the primary MRI reading and central review in Skåne and Västra Götaland

	Skåne					Västra Götaland					All				
PI-RADS score (*n*)	Central review														
					Total					Total					Total
Primary reading	1–2	3	4	5		1–2	3	4	5		1–2	3	4	5	
1–2	97	7	2	2	108	130	10	3	0	143	227	17	5	2	251
3	31	12	3	0	46	23	21	5	3	43	54	24	8	3	89
4	14	9	16	2	41	1	1	8	1	11	15	10	24	3	52
5	3	0	0	12	15	0	1	0	8	9	3	1	0	20	24
Total	145	28	21	16	210	154	24	16	12	206	299	52	37	28	416

MRI = magnetic resonance imaging; PI-RADS = Prostate Imaging Reporting and Data System.

The numbers of scans needed to review to add one biopsy by upgrading to positive (PI-RADS 4–5) MRI in Skåne and Västra Götaland were 23 (nine/210) and 17 (12/206), respectively. Two scans (from Skåne) were upgraded from PI-RADS 2 to PI-RADS 5; both men had a high PSA density and consequently a systematic biopsy that detected GG 2 cancer.

The numbers of scans needed to review to avoid one biopsy (if PSA density <0.15 ng/ml^2^) by downgrading to negative MRI (PI-RADS 1–3) in Skåne and Västra Götaland were 8 (26/210) and 69 (three/206), respectively. Three scans were downgraded from PI-RADS 5 to PI-RADS 2, all from Skåne. The biopsies from these three lesions were benign in two men and showed low-volume GG 2 cancer with <1% Gleason pattern 4 in one man.

The primary reading yielded 76 cases with PI-RADS 4–5 (Table 5). All these 76 men were biopsied: 24 had benign biopsy, nine had GG 1, and 43 had GG 2–5. The biopsy PPV for GG 2–5 was 0.57 (95% CI 0.45–0.67, NNB 1.8). At the central review, 65 scans were scored PI-RADS 4–5. Four men with PI-RADS 4 according to the central review were not biopsied as the primary read was PI-RADS 2–3 with a low PSA density. Out of 61 men biopsied, four had benign biopsy, seven had GG 1, and 50 had GG 2–5. The biopsy PPV for GG 2–5 was 0.82 (95% CI 0.71–0.90), which corresponds to an NNB of 1.2 to detect one case of GG 2–5 cancer. The PPV difference was statistically significant (*p* < 0.001).

**Table 5:** Number of cancer cases detected and positive predictive values by biopsy indication group for the primary MRI reading versus central review

	Primary reading			Central review a		
Biopsy group	PI-RADS ≥4	PI-RADS 3 and PSAD ≥0.15	PI-RADS 1–2 and PSAD ≥0.15	PI-RADS ≥4	PI-RADS 3 and PSAD ≥0.15	PI-RADS 1–2 and PSAD ≥0.15
*n*	76	34	52	65	15	67
Biopsy, *n* (%)	76 (100)	30 (88)	36 (69)	61 (93)	13 (87)	42 (63)
Any PC, *n* (PPV %)	52 (68)	20 (67)	18 (50)	57 (93)	8 (62)	19 (45)
GG 2–5 PC, *n* (PPV %)	43(57)	17 (57)	10 (28)	50 (82)	8 (62)	9 (21)
Number of men needed to biopsy to detect one GG 2–5 PC	1.8	1.8	3.6	1.2	1.6	4.7

GG = grade group; MRI = magnetic resonance imaging; PC = prostate cancer; PI-RADS = Prostate Imaging Reporting and Data System; PPV = positive predictive value; PSAD = prostate-specific antigen density.

a No additional biopsies were taken based on the central review findings.

### 3.3 Sensitivity analyses excluding central review radiologists and in 50-yr-old men only

A sensitivity analysis excluding scans from the centers where the central review radiologists were working showed a similar difference in PPV for positive MRI to detect GG 2–5 PC: primary reading PPV 0.57 and central review PPV 0.83 (*p* < 0.001; Supplementary Table 1).

As some men in Skåne were older than 50 yr, we performed a sensitivity analysis (Table 1 and Supplementary Table 2) including 50-yr-old men only (*n* = 311). This analysis gave similar results as the main analysis, with a greater proportion of PI-RADS 4 lesions at the primary read in Skåne than in Västra Götaland: 18% (19/105) versus 5.3% (11/206). This difference disappeared after the central review: 7.6% (eight/105) versus 7.8% (16/206).

### 3.4 Intraregional variation of primary reading

A detailed analysis of PI-RADS 4 assessments revealed differences across centers (Supplementary Table 3). The largest center had a higher proportion of PI-RADS 4 lesions (26/108 cases, 24%) and a high proportion of benign biopsy results (17/26 cases, 65%). This center read slightly over half of all MRI scans in Skåne.

## 4 Discussion

This first report on MRI assessment and biopsy outcomes with and without a central review in two large, population-based, regional Swedish OPT programs revealed substantial inter- and intraregional differences and highlighted the challenges with aligning diagnostic outcomes for multiple centers.

Overall, the central review significantly increased the PPV of PI-RADS 4–5 to detect GG 2–5 cancer, from 57% to 82%. This translates into a 31% reduction of the NNB. Previous studies have shown that the use of expert radiologists improves diagnostic outcomes. In a clinical study, a central review of prostate MRI scans in men referred to a community hospital almost doubled the PPV for GG 2–5 PC [10]. In the PROBASE screening trial, the central review increased the PPV for GG 2–5 PC from 45% at the primary reading to 72%. PROBASE included comparatively young men (age 45 yr) at the first screen, which may explain a high proportion of indeterminate MRI findings that likely contributed to the results [11]. In our study, most men were 50 yr old, the commonly recommended age to start testing [12], and the radiologists used structured reporting with biopsy feedback and had access to regular diagnostic forums with feedback from discordant cases intended to reduce variability. Despite these measures to aid the primary readers, the central review disagreed with the primary reports in almost one-third of cases.

It appears that the challenges of reading screening MRI should not be underestimated if the promising results in from ongoing screening trials are to be replicated in population-based routine settings. In the Göteborg-2 trial, after a median of 4 yr of screening, 60% of men in the experimental arm avoided biopsy, overdiagnosis of low-risk cancer was reduced by 57%, and few advanced cancer cases were detected at follow-up screens or as interval cancer cases [13]. It is important to notice that the Göteborg-2 trial has a small team of dedicated radiologists, urologists, and pathologists, which contrasts to the decentralized diagnostic chain in Swedish OPT and, most likely, in future nationwide screening programs. The optimal level of centralization of MRI and biopsy in population-based screening is unclear. Limited access to subspecialists and long distances to a tertiary center may favor the use of local resources. On the contrary, the high ethical requirement of not to harm healthy individuals when actively inviting them favors a high level of expertise.

The reported proportions of positive MRI scans and corresponding GG 2–5 cancer detection rates vary between screening studies [11,[14], [15], [16]]. Our study revealed a concerning variability in PI-RADS 4 assignment, both between the two regions and within one of the regions. Despite this, the NNB to find one case of GG 2–5 cancer in men with PI-RADS 4–5 was similar in both regions. Differences in biopsy sampling and pathology assessments may contribute to these findings, but also random effects owing to the small sample size. The finding that almost one-third of PI-RADS 4 lesions from the most discordant center were downgraded at the central review suggests a need for detailed monitoring of results from diagnostic centers and individual radiologists.

The proportion of PI-RADS 1–2 lesions at the primary read in our study was comparable with what has been reported from the Göteborg-2, Stockholm3-MRI, and ProScreen trials, around 60%. Despite that most men participating in the expert read studies Göteborg-2 and Stockholm3-MRI had negative first-round MRI [14,16], few were diagnosed with cancer at follow-up [17]. In our study, only two additional intermediate-risk cancer cases were identified correctly in a review of men with prior negative MRI. Notably, four out of five men with negative MRI in the review were free of clinically important cancer at biopsy, even if their PSA density was high. Future follow-up will show whether these men remain MRI negative and cancer free.

Effective and equitable population-based screening requires a robust diagnostic framework. The structure of breast cancer screening in Sweden, with dedicated centers, specialized diagnosticians, and double reading, serves as a model [18]. Recently, artificial intelligence (AI) models have been integrated into clinical practice as one of two mammogram readers [19]. In prostate MRI, AI has shown potential to reduce inter-reader variability and improve specificity [20,21]. However, the utility of AI in screening cohorts is still not well understood [22], and a pilot study suggests potential challenges [23]. We are currently evaluating the performance of established AI models in screening.

The main strength of this study is the extensive central review in two population-based OPT programs with multicenter MRI scanning.

There were some limitations. One is that the central review was performed by two radiologists only. They served as the primary readers at two of the centers, but the results for PPV did not change when these centers were excluded from a sensitivity analysis. The extended time between the initial reading and central review, random presentation of cases to the central reviewers, and that the reviewers were blinded to the original report, any image annotations, and referral information should minimize the risk of recognition and bias in the review process. Another limitation is that additional biopsies were not taken based on the central MRI review, nor were biopsies taken in men with unsuspicious MRI and a low PSA density.

## 5 Conclusions

In population-based PC screening with local MRI reading, MRI assignment varied across and within regions. Frequent and detailed monitoring of diagnostic outcomes is needed to expose such differences. Centralized MRI reading reduced these and increased the biopsy PPV for GG 2–5 cancer.

  ***Author contributions*:** Jonas Wallström had full access to all the data in the study and takes responsibility for the integrity of the data and the accuracy of the data analysis.

  *Study concept and design*: Wallström, Thimansson.

*Acquisition of data*: Wallström, Thimansson, Bratt, Alterbeck, Godtman, Jiborn.

*Analysis and interpretation of data*: Wallström, Thimansson, Bratt.

*Drafting of the manuscript*: Wallström, Thimansson.

*Critical revision of the manuscript for important intellectual content*: Wallström, Thimansson, Bratt, Alterbeck, Godtman, Jiborn.

*Statistical analysis*: Wallström, Thimansson.

*Obtaining funding*: Wallström, Thimansson.

*Administrative, technical, or material support*: Bratt, Jiborn.

*Supervision*: None.

*Other*: None.

  ***Financial disclosures:*** Jonas Wallström certifies that all conflicts of interest, including specific financial interests and relationships and affiliations relevant to the subject matter or materials discussed in the manuscript (eg, employment/affiliation, grants or funding, consultancies, honoraria, stock ownership or options, expert testimony, royalties, or patents filed, received, or pending), are the following: Rebecka Arnsrud Godtman has received lecture fees and travel honoraria from Bayer and Ipsen.

  ***Funding/Support and role of the sponsor*:** This study was financed by grants from Göteborgs Läkaresällskap (GLS-935760), the Swedish Prostate Cancer Association, and the Swedish state under the agreement between the Swedish Government and the county councils, the ALF agreement (ALFGBG-965228), and Region Västra Götaland (VGFOUREG-993861).

  ***Acknowledgments:*** This study was made possible by the work of the regional OPT-offices: Ann-Karin Börjedal, Ann Carlström, and Sigrid Bengtsson. Marianne Månsson kindly provided statistical expertise.
