# Supplementary material for: A Comparison of Magnetic Resonance Imaging Assessment and Biopsy Outcomes with and Without Central Review in Two Swedish Regional Organized Prostate Cancer Testing Programs
Source: Eur Urol Open Sci. 2025 Jun 5;77:32–8. doi: 10.1016/j.euros.2025.05.008 (PMC12173033; doi:10.1016/j.euros.2025.05.008)
Supplement: Supplementary Data 1 [file mmc1.docx]

**Supplementary material**

Supplementary Table 1: Sensitivity analysis. All MRI scans from the two centers where the centrally reviewing radiologists were also primary readers excluded.


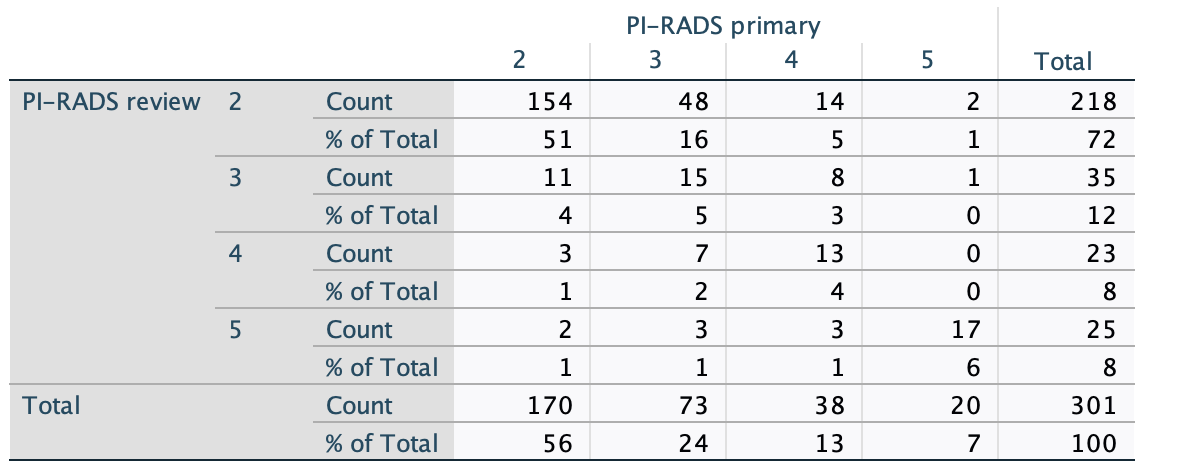


301 cases (115 cases excluded).

Agreement 199/310=66% (versus 295/416=70.9%)

PPV PRIMARY PI-RADS 4-5 GG2-5: 33/58=0.57 (95% CI 0.44-0.69)

PPV REVIEW PI-RADS 4-5: GG2-5: 40/48=0.83(95% CI 0.70-0.91)

PPV difference GG2-5: p-value = <0.001


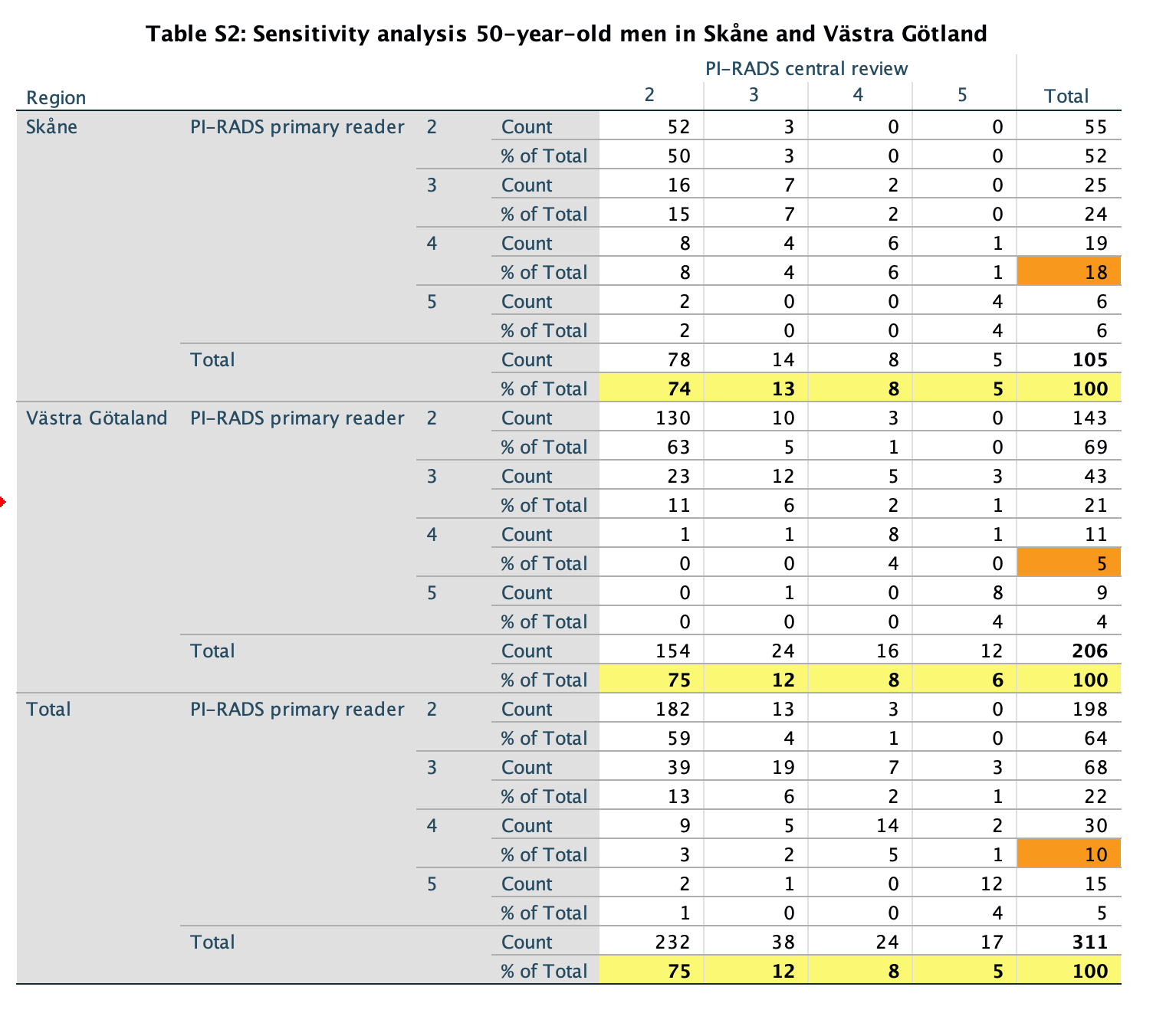


**Supplementary Table 2**

Supplementary Table 3a. Proportions of MRI scans assigned PI-RADS 4 at the different centers in Skåne.

| Center | MRI, n | PI-RADS 4, n (%) | Benign biopsy, n (%) |
| --- | --- | --- | --- |
| 1 | 108 | 26 (24) | 17 (65) |
| 2 | 69 | 12 (17) | 2 (17) |
| 3 | 33 | 3 (9) | 1 (33) |
| Total | 210 | 41 | 20 |

Supplementary Table 3b. Proportions of MRI scans assigned PI-RADS 4 at different centers in Västra Götaland

| Center | MRI, n | PI-RADS 4, n (%) | Benign biopsy, n (%) |
| --- | --- | --- | --- |
| 1 | 77 | 6 (8) | 1 (17) |
| 2 | 68 | 3 (4) | 0 (0) |
| 3-6 | 61 | 2 (3) | 0 (0) |
| Total | 206 | 11 | 1 |

Supplementary Table 4. Age and PSAD of all men and men with lesions up- or downgraded at central review

|  | N | Age (median, IQR) | PSAD (median, IQR) |  |
| --- | --- | --- | --- | --- |
| All | 416 | 50 (50-56) | 0.11 (0.09-0.15) |  |
| Upgraded to PI-RADS ≥4 | 18 | 50 (50-56) | 0.20 (0.085-0.26) |  |
| Downgraded to PI-RADS ≤3 | 29 | 50 (50-56) | 0.12 (0.10-0.17) |  |

**Statistics supplement:**

Supplementary details on bootstrapping procedure:

We studied MRI assessment and detection of GG2-5 prostate cancer before and after central review. A positive MRI was defined as PI-RADS 4-5. P-values for the comparison of PPVs were based on 10,000 bootstrap samples, considering the dependence between PPV estimates due to both primary and central reading were performed on the same men.

The bootstrap procedure was performed in the following steps:

1. 10,000 boot samples were drawn by sampling the rows of the original data set with replacement.
2. For each bootstrap sample:
   - 1. The rows (individuals in sample) where either the primary or central reading (not both) were MRI positive (PI-RADS 4-5) were identified.
     2. Under the null hypothesis, it is equally likely that the primary or central reading is the one with positive MRI; therefore the positive MRI´s were randomly distributed between primary and central reading (Bernoulli, p = 0.5 in each case).
   1. The number of positive MRI´s for the primary reading was counted (a sum of the cases where both readings were MRI positive and those where only the primary was MRI positive (after the shuffling in the previous step). Similarly for the central reading.
   2. The PPV was calculated as the proportion of ISUP >=2 among the positive MRI´s for the primary and central readings, respectively.
   3. The difference in PPV was calculated.
3. The 10,000 differences based on the bootstrap samples form the empirical distribution of difference in PPV under the null hypothesis.
4. The observed difference is compared to the empirical null distribution and the p-value calculated as the proportion of the bootstrap sample differences that are at least as large as the observed one, multiplied by two since the test was two-sided.
